# Impact of the Interruption Duration on Photoluminescence Properties of MOCVD-Grown GaAsP/InAlGaAs Quantum Well Structures

**DOI:** 10.3390/nano14181469

**Published:** 2024-09-10

**Authors:** Bin Wang, Yugang Zeng, Xuezhe Yu, Weijie Gao, Wei Chen, Haoyu Shen, Li Qin, Yongqiang Ning, Lijun Wang

**Affiliations:** 1 Key Laboratory of Luminescence Science and Technology, Chinese Academy of Sciences & State Key Laboratory of Luminescence Science and Applications, Changchun Institute of Optics, Fine Mechanics and Physics, Chinese Academy of Sciences, Changchun 130033, China; wangbin201@mails.ucas.ac.cn (B.W.); yuxuezhe@ciomp.ac.cn (X.Y.); gaoweijie21@mails.ucas.ac.cn (W.G.); chenwei1@ciomp.ac.cn (W.C.); shenhaoyu22@mails.ucas.ac.cn (H.S.); qinl@ciomp.ac.cn (L.Q.); ningyq@ciomp.ac.cn (Y.N.); wanglj@ciomp.ac.cn (L.W.); 2 University of Chinese Academy of Sciences, Beijing 100049, China

**Keywords:** metal–organic chemical vapor deposition, tensile-strained quantum well, GaAsP/InAlGaAs heterostructure, growth interruption time, temperature-dependent photoluminescence

## Abstract

The growth interruption technology is introduced to the growth of GaAsP/InAlGaAs quantum well (QW) structure using metal–organic chemical vapor deposition (MOCVD). The effect of growth interruption time (GIT) on the crystalline quality and optical properties are investigated. The two distinctive emission peaks are the transition recombination between the electron level of conduction band and the light and heavy hole level of valence band in the photoluminescence (PL) at room temperature. The PL peaks present a redshift and merge together with the increasing GIT, which is attributed to the QW energy level shift caused by the increase in arsenic concentrations in GaAsP QW, the diversified thickness of QW and the variations of indium components in the InAlGaAs barrier layer. The Gaussian deconvolution parameters in temperature-dependent PL (TDPL) show that the GaAsP/InAlGaAs QW with a GIT of 6 s has a 565.74 meV activation energy, enhancing the carrier confinement in QW and the PL intensity, while the 6 s-GIT GaAsP QW has the increasing interface roughness and the non-radiative centers at the InGaAsP intermediate layer, leading to a spectral broadening. The QW with 10 s-GIT exhibits a small full width at half maximum (FWHM) with the various temperature, indicating reduced interface roughness and excellent crystal quality. An increase in GIT may be suitable for optimizing the optical properties of GaAsP/InAlGaAs QW.

## 1. Introduction

Visible semiconductor lasers are extensively utilized in optical scanning and printing applications. They also serve as pumping light sources for solid-state lasers [1,2]. AlGaAs is extensively studied among III-V semiconductor alloys due to its nearly identical lattice to GaAs and facilitating high-quality growth. Lasers utilizing AlGaAs-based quantum wells (QWs) as active regions typically operate in the 700–780 nm range through modulations of Al composition. However, AlGaAs QW lasers operating below 780 nm degrade due to the high Al content in the active region [3]. Furthermore, AlGaAs transitions into an indirect band-gap semiconductor when the Al concentration exceeds 0.43, thus making the strategy for shortening emission wavelengths ineffective [4]. The (Al)GaInP material system has been extensively studied in visible light emitting lasers [5,6,7]. However, controlling the lattice constant to match the GaAs substrate is challenging, and achieving a high p-doped level in the cladding layers is difficult. The growth of the (Al)GaInP material system typically occurs on a large misoriented GaAs substrate, which is miscut from the [100] direction towards the [111] direction to suppress the spontaneously ordering phase on the {111} planes during growth [5]. This approach is incompatible with standard laser manufacture on the (100) substrate.

Compressively strained InAlGaAs active regions have also been utilized for laser structures operating in the red visible light region. The presence of indium reduces dark-line defects, thereby making the laser less sensitive to defects induced during fabrication. Lasers containing compressively strained active regions have achieved lower threshold currents compared to the lattice-matched AlGaAs-active regions. However, the subtle carrier confinement due to small band offset in InAlGaAs/GaAs material systems leads to the temperature sensitivity in the device, limiting its high-power output. Moreover, the increased possibility of catastrophic optical damage due to higher Al content in the active layer deteriorates the reliability of lasers [8]. Al-free material systems such as InGaAsP/AlGaInP have been used to achieve emission in the 700–780 nm region, outperforming their InAlGaAs counterparts in high-power output and device operation stability [9,10]. Developing InGaAsP is more challenging to control than its predecessors due to lattice mismatch with GaAs and poor heterostructure interface. The shortcomings of quaternary alloy InGaAsP include difficulties in precisely controlling compositions during growth and the presence of a wider miscibility gap in the compositions lattice-matched to GaAs. The inhomogeneous growth of InGaAsP frequently results in decomposition into InAs or GaP regions, leading to phase separation that degrades crystal quality and impairs laser performance [11,12,13,14].

Tensile strain enables the laser emitting in the transverse magnetic (TM) mode, offering a high differential modal gain [3,15]. Tensile-strained GaAsP QW embedded in AlGaAs has been widely used for lasers ranging from 700 to 780 nm in wavelength [3,16,17,18,19]. The higher conduction band offset effectively inhibits carrier thermal escape from the active region, making it well-suited for high power output and conversion efficiency in GaAsP-active lasers. Recently, Wang et al. reported a maximum efficiency of 71% in a GaAsP QW laser [20]. Although the high band offset partly decreases the carrier escape from the QW, many electrons penetrate into the waveguide or even the cladding layers. The tensile-strained GaAsP QW directly encapsulated in the AlGaAs waveguide may produce certain defects and introduce non-recombination centers at the interface. The compressively compensated barrier layer can mitigate the negative impacts of the tensile-strained QW. The InAlGaAs layer with a low indium concentration can alleviate the effects of tensile strain from the GaAsP QW, thereby avoiding the detrimental effects of lattice distortion. The increased conduction band offset enhances the electron confinement ability in the GaAsP/InAlGaAs/AlGaAs structure. However, introducing an InAlGaAS barrier layer leads to As-P exchange at the interface, resulting in roughness and compositional fluctuation. Growth interruption in MOCVD has been proven effective in improving interface flatness and crystal quality [21,22,23,24]. No reports exist on how the growth interruption time (GIT) affects the GaAsP/InAlGaAs interface.

In this study, single GaAsP/InAlGaAs quantum well structures were grown by MOCVD at different growth interruption times (GITs). The correlation between GITs and the optical properties of GaAsP/InAlGaAs QW structures was characterized using photoluminescence (PL) under room temperature (RT) conditions with varying excitation power, high-resolution X-ray diffraction (HRXRD), and temperature-dependent photoluminescence (TDPL). The two wavelength peaks in the GaAsP QW corresponded to transitions between the first electron level in the conduction band and the first light and heavy hole levels in the valence band. The spectral redshift observed with increasing GITs was attributed to variations in As concentration, changes in QW thickness, and the III-group contents in the InAlGaAs barrier. The TDPL spectra were deconvoluted to two Gaussian fitting components. The parameters of Gaussian parameters corresponding to electron-to-light-hole recombination can be well-fitted. Samples with a 6 s-GIT exhibited a maximum activation energy of 565.74 meV, demonstrating improved carrier confinement in the GaAsP QW. Formation of the InGaAsP intermediate layer introduced non-radiative centers, increased the interface roughness, and broadened the PL full width at half maximum (FWHM) for the 6 s-GIT sample. The increased thickness of the InGaAsP interlayer resulted in a flattened interface in the 10 s-GIT sample. The FWHM of Gaussian fitting results suggested that GaAsP/InAlGaAs grown with a 10 s-GIT improved the QW interface quality and reduced the presence of the non-radiative centers. This study reveals that adjusting GIT during MOCVD has the potential to improve interface quality and enhance recombination intensity, thereby providing valuable insights for optimizing the optical properties of QW lasers.

## 2. Materials and Methods

**Figure 1:**
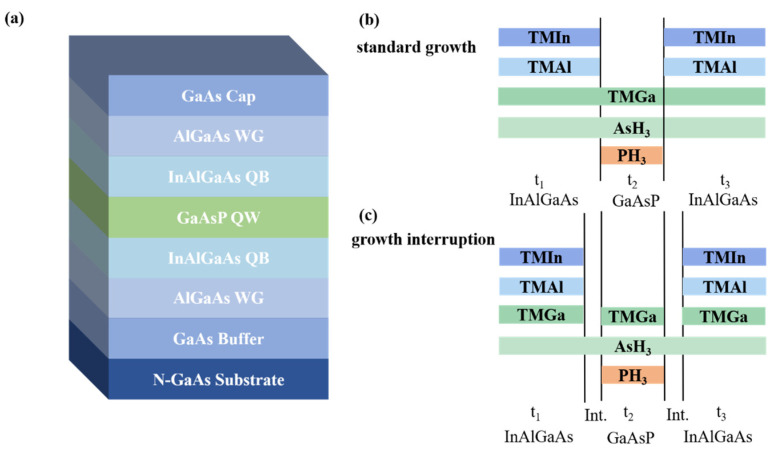
(**a**) Schematic of the GaAsP/InAlGaAs structure. (**b**) Schematic of standard growth and (**c**) growth interruption process in GaAsP/InAlGaAs QW, t_1_, t_2_, and t_3_ represent the growth process of lower InAlGaAs barrier, GaAsP QW, and upper InAlGaAs barrier. “Int.” denotes the growth interruption between the GaAsP and InAlGaAs layers. The colored bar indicates the corresponding precursor flow; otherwise, the precursor source is disabled.

The GaAsP/InAlGaAs structures with varying interruption times were grown using low-pressure metal–organic chemical vapor deposition (LP-MOCVD) in a horizontal MOCVD system (AIXTRON 200/4). The III-troup reactive precursors were trimethylgallium (TMGa), trimethylindium (TMIn), and trimethylaluminum (TMAl), and the V-group precursors were arsine (AsH_3_) and phosphine (PH_3_), respectively. Ultra-high-purity hydrogen (H_2_) was used as a carrier gas. The epitaxial structure was grown on semi-insulating GaAs(100) substrates with 0° misorientation. The specific structure is illustrated in Figure 1a. The detailed growth process was as follows. Before the growth of the epitaxial structure, a 5 min high-temperature baking was performed at 710 °C to remove oxides from the substrate surface under an AsH_3_ protective atmosphere. The reactor temperature was fixed at 710 °C in the whole epitaxial process. During the growth process, the AsH_3_ and PH_3_ flow values were 80 sccm and 250 sccm, respectively. A 200 nm GaAs buffer layer was first formed on the GaAs substrate by continuous injection of TMGa and AsH_3_ to achieve a flat growth surface. Then, a 200 nm Al_0.5_Ga_0.5_As lower cladding layer was grown, followed by the formation of the InAlGaAs barrier layer. There were varying GITs before and after the formation of the GaAsP QW layer. Subsequently, the symmetrical structure of the InAlGaAs barrier and the Al_0.5_Ga_0.5_As upper cladding layers was formed sequentially. Finally, a 50 nm GaAs capping layer was deposited at the top of the epitaxial structure. The indium introduced by the InAlGaAs barrier layer was used to compensate for the tensile strain from the GaAsP QW, with conjunction of aluminum component to offer a large conduction band offset to confine the electrons within QW.

Growth interruption in MOCVD is recognized as an efficient method for improving the quality of the heterojunction interface and reducing the density of nonradiative centers [25]. To achieve a stable flow of group V precursors and a smooth interface between GaAsP and InAlGaAs, growth interruption was applied before and after the growth of GaAsP QW. Figure 1b,c schematically illustrate the typical standard growth and growth interruption processes for fabricating GaAsP/InAlGaAs using MOCVD. During the formation process of the InAlGaAs barrier layer, the TMGa, TMIn, TMAl, and AsH_3_ source valves were turned on simultaneously. Unlike the continuous injection of TMGa in the standard growth, the TMGa source was shut off while the AsH_3_ source remained injected during the growth interruption. The AsH_3_ source remained open to prevent the desorption of the As atoms from the grown surface and to achieve a smooth crystal surface. Samples with interruption times of 0 s, 4 s, 6 s, and 10 s are referred to as samples A, B, C, and D, respectively. The growth conditions, except for the GIT, remained constant.

The structural quality and optical properties of the epitaxial layers were investigated using high-resolution X-ray diffraction (HRXRD, PANalytical B.V, Tokyo, Japan) omega-2theta scanning of the (004) planes and photoluminescence (PL) at room temperature. The HRXRD rocking curve scan utilized Cu Kα X-ray with a wavelength of 1.54059 Å. The PL light source had a wavelength of 532 nm, and the output power varied from 5 mW to 110 mW. To understand the luminescence mechanism of GaAsP/InAlGaAs and the interface improvement caused by growth interruption, temperature-dependence PL (FL920, Edinburgh Instruments Ltd., Livingston, UK) measurements were performed in a variable temperature ranging from 77 to 300 K. The light source was an Xe lamp providing a 495 nm excitation wavelength. The PL emission from the sample was dispersed by a single grating spectrometer and detected by an infrared spectral camera after amplification by a photomultiplier tube (PMT, Hamamatsu Photonics, Shizuoka, Japan).

## 3. Results and Discussion

### 3.1. Photoluminescence at Room Temperature

**Figure 2:**
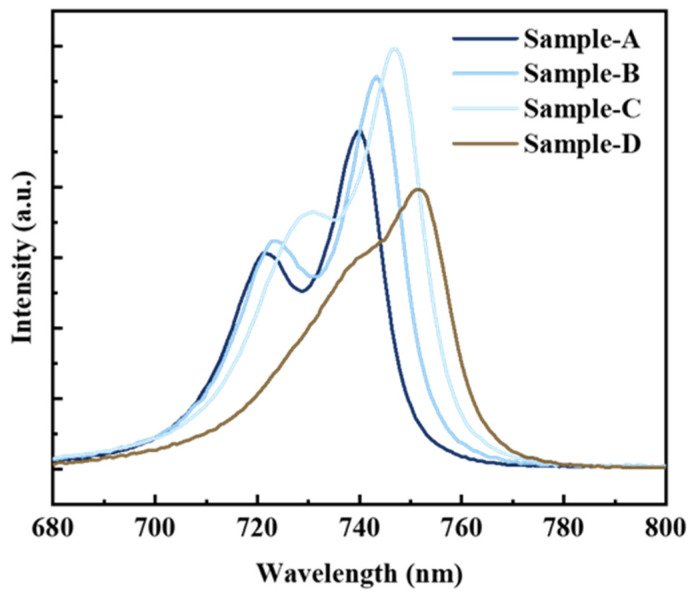
PL spectra of Samples A–D at room temperature.

Figure 2 presents the PL results of samples with different GITs at room temperature. Two emission peaks were observed for each sample, and a spectral redshift occurred with increasing GIT. The high-energy emission peak gradually shifted and merged with the lower-energy peak as the interruption time increased. An enhancement in PL intensity with increasing GIT was observed, indicating that an appropriately increased GIT can facilitate the radiative recombination of QW. When the GIT reached 10 s, the GaAsP/InAlGaAs PL intensity declined. For simplicity, the low-energy (long-wavelength) and high-energy (short-wavelength) peaks are referred to as Peak 1 and Peak 2, respectively, in the following text. Sample C, with a 6 s interruption time, had the maximum PL intensity. A high-quality QW exhibits strong emissive intensity.

**Figure 3:**
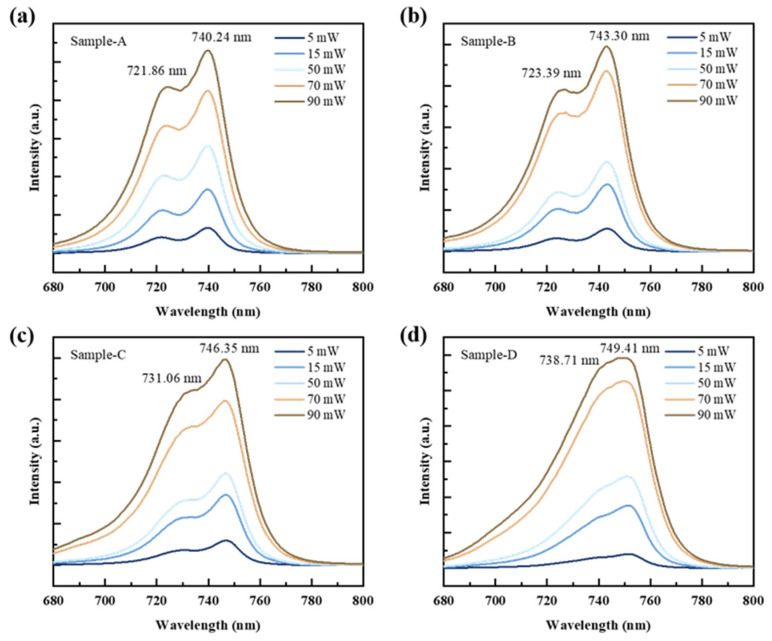
Excitation-power-dependent PL spectra ranging from 5 mW to 90 mW for GaAsP/InAlGaAs structures. (**a**) Sample-A, (**b**) Sample-B, (**c**) Sample-C, (**d**) Sample-D.

To confirm the source of the two peaks and the spectral redshift in Figure 2, excitation-power-dependent PL spectra ranging from 5 mW to 90 mW were obtained and are presented in Figure 3. It was observed that both Peak 1 and Peak 2 in samples A–D remained unchanged with increasing excitation power. The wavelengths of Peak 1 for samples A–D were 740.24 nm, 743.30 nm, 746.35 nm, and 749.41 nm, respectively. The corresponding wavelengths of Peak 2 were 721.86 nm, 723.39 nm, 731.06 nm, and 738.71 nm.

### 3.2. The Simulation of Quantum Well Subband Levels and Analysis of RT-PL

The anisotropic parabolic approximation method was used to calculate the quantum levels at the Γ point using PICS3D. The active region parameters were carefully adjusted, and the results were almost consistent with the experimental PL spectra. The detailed calculated results are presented in Table 1. The relative positions of the two peaks depend on the QW layer, including the thickness and content of compound alloys, and the concentration of quantum barrier layers. The thickness of the quantum barrier layer was maintained at 4 nm. According to Table 1, Peaks 1 and 2 in the PL spectra were attributed to the intrinsic–exciton transition recombination of the electron-light hole (e-lh1) and electron-heavy hole (e-hh1), respectively. The subtle differences in emission wavelength between the experiments and simulations can be attributed to residual strain, imprecise material systems parameters, and the convergence step in software. The As concentration of sample C was about 77% and the emission wavelength of e-lh1 in sample C was 746.18 nm, almost consistent with reports [26]. Calculating the quantum energy level of the active region structure is a better way to explain the two peaks in the PL test and analyze the element intermixing. The simulated results are inevitably slightly different from the measurement, but they provide guidance for the qualitative analysis of the interface process.

The As concentration in the GaAsP QW increased from 73% to 78.5% as the GITs varied from 0 s to 10 s. The V-group gas memory effect in the MOCVD growth system led to the incorporation of As into the GaAsP QW at an almost constant concentration [27]. At the first interface of GaAsP QW, the continuous injection of AsH_3_ reacted with the incoming PH_3_ to form the GaAsP QW. The residual AsH_3_ was incorporated into the GaAsP QW at a constant concentration, increasing the As content of GaAsP. Another result of memory effects was the formation of a composition gradient transition layer at the adjacent interfaces [28,29]. During the interruption time, the surface was covered with As multilayers with variable stoichiometries; research shows that As-stabilized surfaces terminate in a tetramer or dimer using LP-MOCVD [30]. After the formation of the first InAlGaAs barrier layer, the As-rich surface formed a several-monolayer (ML) thickness of GaAsP when the PH_3_ was switched on. At the second GaAsP QW interface, the As adatoms reacted with the GaAsP growth front to form a GaAsP layer with molecular thickness, making Sample B thicker than Sample A. When the interruption time exceeded 4 s, the As-stabilized surface binding sites were saturated, making it difficult to adsorb As atoms at the QW interfaces. The As-P exchange at the QW interfaces reduced the QW thickness. The tensile strain split the light and heavy hole eigenenergies, resulting in the observation of two peak wavelengths in the PL results of Samples A, B, and C. The splitting gap between the two peaks gradually decreased with increasing interruption time. The first-light and heavy-hole energy bands converged, resulting in a single combined PL peak. The thin QW pulled the light and heavy hole eigenenergies back together due to the quantum size effect, causing the two peaks to merge into a single PL peak, as seen in Sample D in Figure 2. The merging phenomenon of light and heavy-hole level shown by the calculation due to the decreased QW thickness was consistent with previous reports [31,32]. The calculated results showed an increase in Ga and Al concentration in the InAlGaAs quantum barrier layer, attributed to the formation of a quaternary InGaAsP intermediate layer during V-gas switching [33]. The PH_3_ pyrolysis products, combined with the efficient As carry-over from the excess As, and the strong As and P carry-over penetrated into the lower InAlGaAs barrier layer, forming a monolayer-thickness quaternary InGaAsP interlayer, with minor content penetrating into the upper InAlGaAs layer to form the quaternary compound [34,35]. Additionally, in the GaAsP/InAlGaAs QW system, P being the smallest element tends to diffuse into the InAlGaAs layer. The InGaAsP intermediate layer may introduce non-radiative centers and broaden the PL FWHM, resulting in the decreased PL intensity of samples A–C, as depicted in Figure 2.

### 3.3. The X-ray Diffraction Results

**Figure 4:**
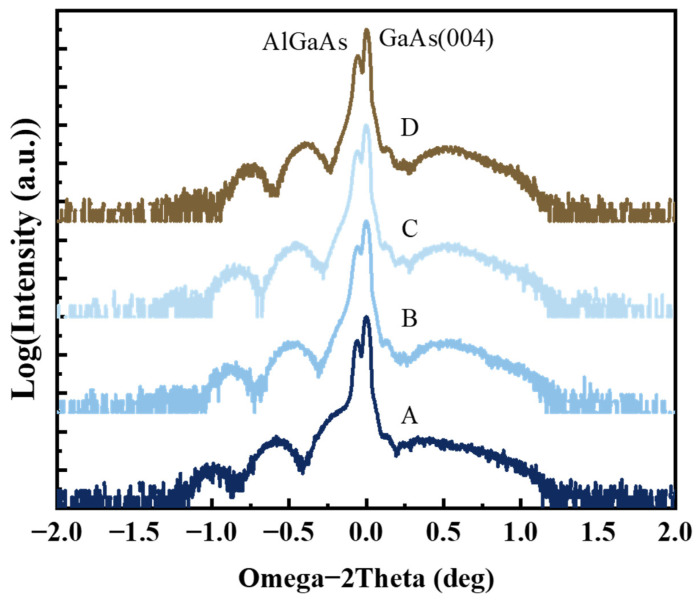
HRXRD Omega-2Theta scan of the GaAsP/InAlGaAs structure around the (004) reflection, conducted with different interruption times. The XRD results of Sample−A, B, C and D are marked with A, B, C and D in the figure. The horizontal scale is referenced from the relative coordinate of the main peak (GaAs (004)). The vertical scales are logarithmic and normalized. For clarity in comparison, the data are plotted with a 0.5-unit offset.

Figure 4 shows a comparison of HRXRD Omega-2Theta scans around the GaAs (004) reflection from the GaAsP/InAlGaAs QW with different interruption times. The horizontal coordinate of the swing curve is relative, with the origin corresponding to the main peak, GaAs (004). The experimental intensities are normalized. The diffraction intensity is logarithmic to better observe the diffraction peaks. The main diffraction peak at 0 degrees corresponds to GaAs (004), with a nearby peak on the left corresponding to the Al_0.5_Ga_0.5_As layer. There are no significant changes among these samples, indicating consistent growth of the Al_0.5_Ga_0.5_As growth. The series of satellite peaks on the left correspond to the InAlGaAs layers. The multiple diffraction peaks result from interference generated by the two InAlGaAs layers. The InAlGaAs peaks shift towards the main peak as the interruption time increases, indicating decreased compressive strain in the InAlGaAs layer. The decreased compressive strain with increasing GITs may be caused by the small In components precipitating form the InAlGaAs barrier layer, forming a quaternary InGaAsP intermediate layer. The InGaAsP diffraction peak is not observed, as the molecular monolayer is too thin for its diffraction intensity to be visible, even at logarithmic XRD intensity. This does not imply the nonexistence of the InGaAsP monolayer, as there is significant work reported on it [36,37,38]. The GaAsP QW diffraction peak is located on the right side of the main peak, and the entire envelope remains almost unchanged with increasing GIT. While the GaAsP diffraction peak of sample A is closer to the substrate peak, it may be attributed to the indium separation and phosphorus diffusion, which leads to the formation of InGaAsP interlayer and reduces the stability of the active region. The uncertainty behavior of the InGaAsP interlayer results in the deviation between XRD results and simulation of Table 1.

### 3.4. The Temperature-Dependent Photoluminescence Test

**Figure 5:**
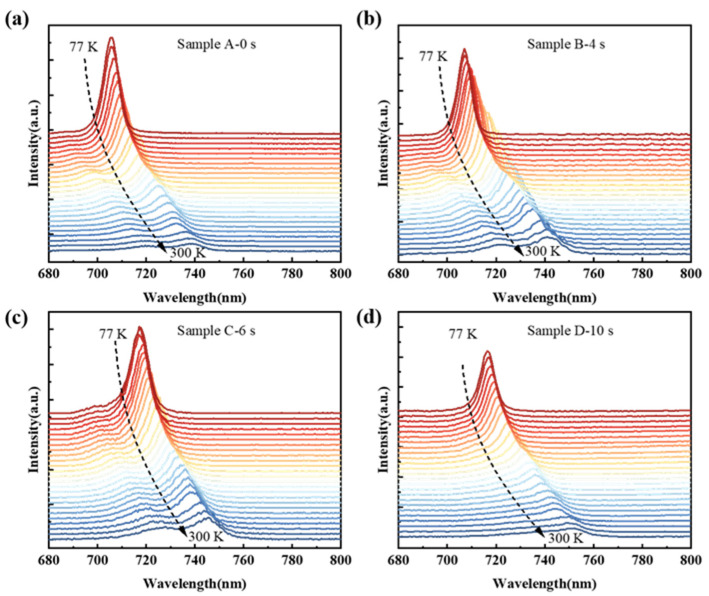
The TDPL spectra from 77 K to 300 K of the GaAsP/InAlGaAs structure with different GITs. The red gradient to blue curve represents from 77 K to 300 K (77 K, 80 K, 90 K, 100 K, 105 K, 115 K, 125 K, 130 K, 140 K, 155 K, 166 K, 180 K, 195 K, 200 K, 205 K, 215 K, 220 K, 235 K, 245 K, 255 K, 260 K, 265 K, 285 K, 295 K and 300 K) TDPL results. (**a**) Sample-A (0 s), (**b**) Sample B (4 s), (**c**) Sample C (6 s), (**d**) Sample D (10 s).

**Figure 6:**
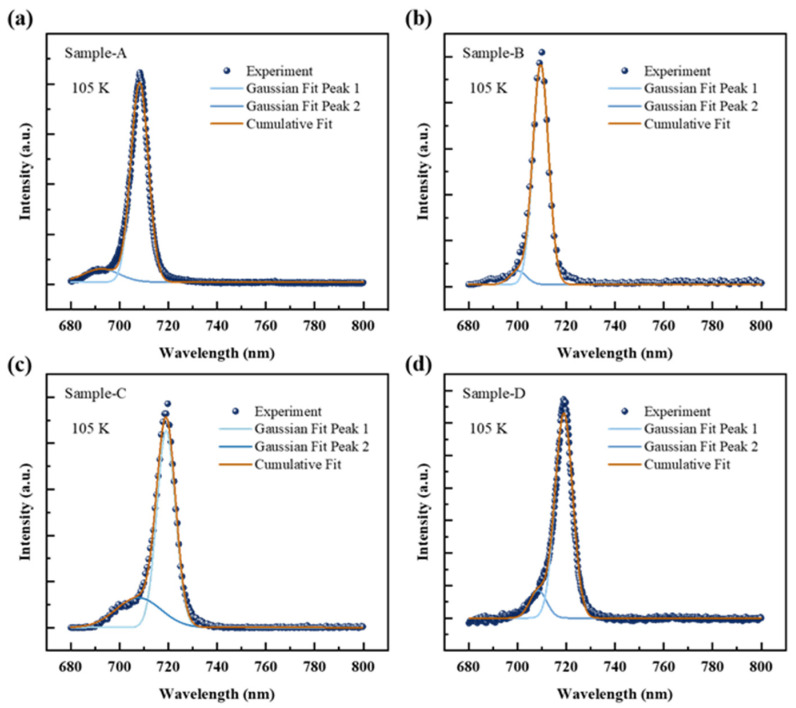
The PL spectra and Gaussian fitting components of the GaAsP/InAlGaAs QW structure with various GITs at 105 K. (**a**) Sample-A (0 s), (**b**) Sample B (4 s), (**c**) Sample C (6 s), (**d**) Sample D (10 s).

To investigate the interface roughness with different GITs and their effects on the optical characteristics of the GaAsP/InAlGaAs, temperature-dependent photoluminescence (TDPL) was conducted. The TDPL results are displayed in Figure 5. The TDPL was performed with temperatures ranging from 77 K to 300 K. Clearly, the PL spectra of Samples A–D exhibit a redshift with increasing temperature. The PL spectra of Samples A, B, and C show two emission peaks: Peak 1 at longer wavelengths and Peak 2 at shorter wavelengths, corresponding to the eigenenergy transitions of e-lh1 and e-hh1 as detailed in Table 1. The TDPL result for Sample D presents a single peak with a high-energy tail extending several tens of nanometers. This tail is attributed to the merge of high-energy heavy hole level and low-energy light hole level. Sample C exhibits two distinctive emission peaks at 77 K, while others show a single peak. High-energy Peak 2 is observed in Samples A and B above 105 K. For interface comparison and identification of energy peaks, PL spectra are deconvoluted using two Gaussian curves. Figure 6 illustrates the schematic diagram of Gaussian deconvolutions at 105 K in different GIT samples. The subsequent discussion of PL parameters is based on the Gaussian fitting components from 77 to 300 K.

**Figure 7:**
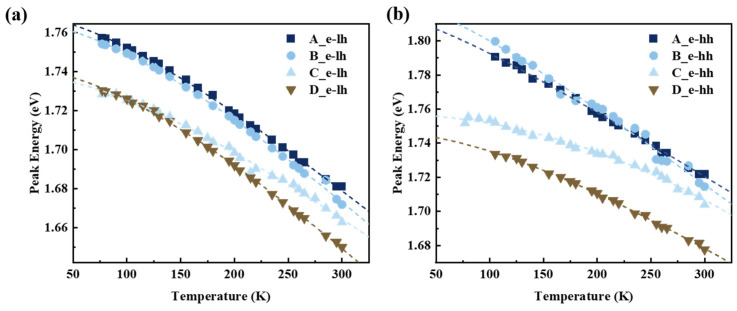
The peak energies of the Gaussian fitting components for (**a**) e-lh1 and (**b**) e-hh1 in GaAsP/InAlGaAs QW structures were presented in the TDPL across a temperature range from 77 K to 300 K.

Figure 7 illustrates the variation of the PL peak energies of e-lh1 and e-hh1 in samples with respect to temperature. The PL peak energy decreases monotonically with increasing temperature. These variations in emissive energy peaks conform well to the Varshni formula [39],

(1) $$E_{g}\left(T\right)=E_{g}\left(0\right)-\frac{\alpha T^{2}}{\beta +T},$$

where *Eg*(*T*) represents the value of the bandgap at temperature *T*, *Eg*(0) is the bandgap energy at 0 K, and *α* and *β* are the Varshni parameters and Debye temperature, respectively. The Varshni formula elucidates two temperature-dependent behaviors: (1) the variation of bandgap with increase in lattice constant due to thermal expansion and (2) the temperature-dependent electron–phonon interactions induced by shifts in conduction and valence bands. The fitting parameters obtained are presented in Table 2. The constant α of e-hh1 exhibits less temperature dependency across the four samples compared to e-lh1, and the small values of α suggest potential for stable temperature performance in the device. The fitted *α* and *β* values are consistent in levels compared to the similar material system InGaAs/GaAsP [40].

**Figure 8:**
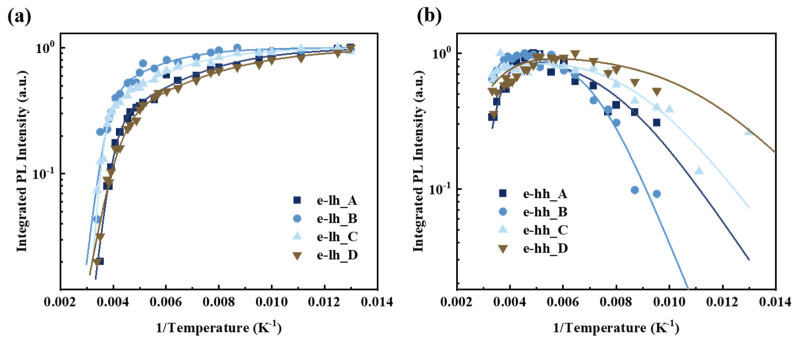
The integrated PL intensities of the Gaussian fitting components for (**a**) e-lh1 and (**b**) e-hh1 in GaAsP/InAlGaAs QW structures with different GITs. The solid lines represent the fitting results using the Arrhenius formula.

To elucidate the quenching mechanism of PL intensities with increasing temperature, the temperature-dependent normalized integrated PL intensities are well-fitted using the two-channel Arrhenius formula [39],

(2) $$I\left(T\right)=\frac{1}{1+C_{1}\exp\left(-\frac{E_{A1}}{k_{B}T}\right)+C_{2}\exp\left(-\frac{E_{A2}}{k_{B}T}\right)},$$

where *I*(*T*) is the integrated PL intensity at temperatures *T*, *k_B_* is the Boltzmann constant, *E_A_*_1_ and *E_A_*_2_ are the activation energies related to nonradiative recombination, and *C*_1_ and *C*_2_ are constants representing the density of nonradiative recombination centers [41]. The integrated PL intensities of e-lh and e-hh Gaussian components at different temperatures and the fitting results are depicted in Figure 8a,b. The integrated PL intensity of e-lh1 is consistent with Equation (2), with detailed fitting results provided in Table 3. The significant reduction in the number of electron-hole pairs leads to PL intensity quenching at elevated temperatures. Two types of nonradiative recombination channels contribute to the alteration of the PL intensity during temperature increase: (1) thermally activated nonradiative recombination in defects induced by the As-P exchange at the GaAsP and InAlGaAs interface [42] and (2) nonradiative recombination of thermal carriers in the InGaAsP interlayer as they escape from the GaAsP QW to the InAlGaAs barrier. The fluctuation in material content of the InGaAsP interlayer results in higher nonradiative recombination densities. The maximum activation energy *E_A_*_1_ of sample C is 565.74 meV, indicating strong quantum confinement in the GaAsP QW, consistent with the PL intensity for Sample C in Figure 2. Conversely, the lower value of *E_A_*_1_ in Sample D contributs to the weaker PL intensity observed in Figure 2. The maximum value of *C*_1_ in Sample C suggests significant non-radiative defect formation by the InGaAsP interlayer at the interface during the GIT. The *C*_1_ value of the sample with a 4 s-GIT is lower than that of the sample without interruption. This reduction is attributed to the inhibition of the negative impact of As-P exchange during the 4 s-GIT by the abundant decomposition of AsH_3_, resulting in a relatively flat growth surface. The *C*_1_ value of the sample with a 6 s-GIT increases sharply, attributed to the introduction of significant defects during the formation of the InGaAsP intermediate layer over a longer GIT. During the QW upper interface growth, substantial TMIn is absorbed on the As-rich GaAsP surface, forming the InGaAsP interlayer. When the GIT ranges from 4 s to 6 s, the thickness of InGaAsP increases with longer interruption times, resulting in higher non-radiative recombination centers. Similarly, the absorbed PH_3_ on the lower As-rich InAlGaAs barrier surface generates the InGaAsP interlayer before the formation of GaAsP QW. Sample D with a 10 s-GIT has the minimum value of *C*_1_, suggesting the flatten interface. During the growth interruption, the thicker InGaAsP interlayer smooths the interface and reduces the density of non-radiative recombination to a certain extent. The quality of the QW heterostructure interface improves with the increasing GIT to a certain extent [43]. The decrease in PL intensity of sample D in Figure 2 may be attributed to the relatively thick InGaAsP layer on both sides of the GaAsP QW. This configuration weakens the confinement ability of the InAlGaAs barrier layer due to the formation of an intermediate energy band between the QW and the barrier layer. Samples A–D exhibit similar values of *C*_2_ and *E_A_*_2_, indicating the same type of nonradiative recombination channel due to the As-P exchange. The small value of *C*_2_ in Sample D also suggests minimal interface roughness.

The Gaussian component of e-hh1 shows poor fitting to Equation (2) in these samples. An abnormal increase is observed in the integrated PL intensity of the e-hh1 in the range of 105–205 K. This phenomenon is attributed to the enhanced carrier mobility at low temperatures, resulting in the migration of photogenerated carriers from barriers to QW under nonresonant excitation [44]. The integrated PL intensity of e-hh1 increases at 105–205 K, suggesting that abundant carriers are captured from the adjacent light-hole level regions to the heavy-hole level in the QW. Initially, carriers generated in the InAlGaAs barriers are captured by the GaAsP QW at low temperatures, diffusing along the GaAsP QW and recombining over there. The integrated PL intensity is enhanced within the temperature range of 105–205 K. As the temperature increases further, carriers thermalize and escape from the well, leading to PL quenching via nonradiative recombination, resulting in a dramatic drop-integrated PL intensity.

**Figure 9:**
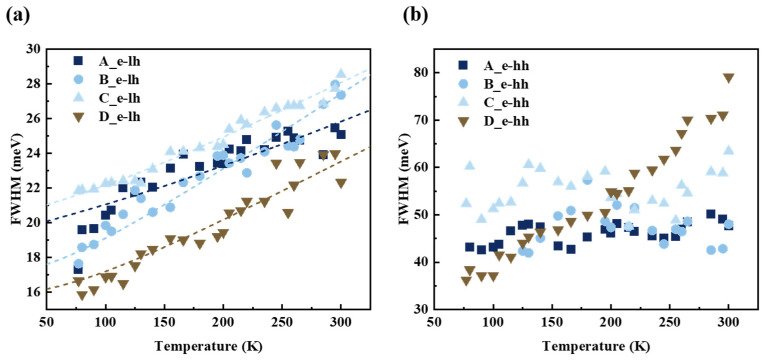
The PL FWHM values of Gaussian fitting components in GaAsP/InAlGaAs QW, (**a**) e-lh1, (**b**) e-hh1.

The interface roughness, crystal quality, and alloy disorder of the GaAsP/InAlGaAs structure are further evaluated using the PL FWHM. The FWHM of the TDPL spectrum is investigated, as depicted in Figure 9. Figure 9a shows the FWHM of e-lh1 in Samples A–D. The FWHM increases monotonically with rising temperature. The electron–phonon interactions are reinforced at high temperatures, which enhances exciton scattering and broadens the PL linewidths. The FWHM of TDPL experimental expression is as follows [45]:

(3) $$\Gamma \left(T\right)=\Gamma_{0}+\sigma T+\frac{\gamma }{\exp\left(\frac{\hbar \omega_{LO}}{k_{B}T}\right)-1},$$

where the Γ(*T*) is the full linewidth of the PL at different temperature *T* and Γ_0_ represents the simplified inhomogeneous broadening due to interface roughness and alloy disorder. The second and third items in Equation (3) are homogeneous contributions to the linewidth. The second item involves exciton interactions with acoustic phonons and *σ* representing the intensity of acoustic phonon scattering. And the third item is interpreted as the mutual effects on the longitudinal optical (LO) phonons, where *γ* is the proportionality factor and the denominator as the Bose function of the thermal phonons. Here, *ħω_LO_* signifies LO phonon energy of the QW layer. Due to uncertainties of the Gaussian decomposition results, Equation (3) cannot fit properly only for e-lh1 (Figure 9a). The dashed lines in Figure 9a illustrate the fitting results for e-lh1. Table 4 presents the fitted values of Γ_0_, *σ*, *γ,* and *ħω_LO_* for Samples A–D.

The Γ_0_ value of 15.599 meV in Sample D is the smallest among these samples, indicating that the extended growth interruption improves the interface roughness and reduces the alloy disorder. Similarly, the *σ* value in Sample D, comparable to that of Sample B, indicates that the thicker InGaAsP interlayer alleviates the phonon scattering with the increase in interruption times. The relative relationships between Γ_0_ and *σ* in Samples A–D is same as that of *C*_1_ in Table 3, confirming that 10 s-GIT can obtain a smooth heterogeneous interface. The *ħω_LO_* value of Sample D is lower than that of Sample C, attributed to the reduced nonradiative activation energies *E_A_*_1_ due to strain modulation at the QW interface from altered QW and QB layer components. Based on the above TDPL results, a GIT value of 6 s enhances the PL intensity at the cost of broadening PL FWHM, whereas a longer 10 s interruption time reduces interfacial roughness and alloy disorder through thick interlayer formation, narrowing the luminescence width in GaAsP/InAlGaAs structure. The InGaAsP interlayer flattens the GaAsP/InAlGaAs interface. Figure 9b shows the FWHM versus temperature of the e-hh1 Gaussian fits. Only a qualitative discussion of interface quality as a function of temperature is possible due to the uncertainties in the results of the Gaussian decomposition. The FWHM of e-hh1 in Samples A, B, and C are relatively fluctuated around a certain constant across the temperature range of 77–300 K. No enhancement in exciton–phonon coupling is observed with increasing temperature. Spectral broadening caused by e-hh1 in Sample C remains considerable. The FWHM of e-hh1 in Sample D shows further broadening of the spectrum with increasing temperature. Broadening of the e-hh1 FWHM in Sample D with increasing temperature is attributed to exciton coupling with acoustic and LO phonons. These results indicate the potential to tune the emission wavelength of QW through various GITs during MOCVD. Compared to TDPL to evaluate the interface condition, the direct measurement method is more intuitive to investigate the effect of GITs on the quality and crystallinity of the interface. TDPL provides a qualitatively way to evaluate heterogeneous interface from the spectral perspective. Further research objectives include exploring the detailed process and mechanism of interfacial composition variations at the heterogeneous interface using direct methods, such as transmission electron microscope or secondary ion mass spectroscopy, as well as interventions through growth modulation.

## 4. Conclusions

In conclusion, the effects of different GITs on optical properties of GaAsP/InAlGaAs QW grown by MOCVD were investigated. Samples with interruption times ranging from 0 s to 6 s exhibited two emission peaks in room temperature PL. The sample with a 10 s interruption time showed merged peaks. The separation between the two emissive peaks in the PL spectra decreased with increasing GIT, attributed to higher As content in the GaAsP QW, variations in QW thickness, and reduced In incorporation in the InAlGaAs barrier layer. HRXRD results indicated a reduction in In concentration in the barrier and the decreased compressive strain. The wavelengths of the two peaks corresponded to transition energies from the first electron energy level to the first-light and heavy-hole energy levels, as predicted by the k·p theory. With a GIT of 6 s, the GaAsP QW exhibited maximum PL intensity at room temperature, attributed to the highest activation energy of 565.74 meV obtained from TDPL (77–300 K). Broadening of the PL spectrum in the sample with a 6 s GIT was attributed to poor interfaces caused by the non-radiative centers in the quaternary InGaAsP interlayer. The thicker quaternary interlayer formed during the longer interruption times reduced interface roughness and improved the PL FWHM, observed in the 10 s GIT due to the flattened InGaAsP interlayer. Temperature-independent recombination between the electron and heavy-hole levels was expected to facilitate the development of relatively temperature-insensitive devices. Analysis of the TDPL spectra in and Gaussian fitting curves in samples with different GITs indicated that a 10 s growth interruption improves the quality of heterogeneous interfaces. Formation of the quaternary interlayer through As-P exchange and diffusion of III-group atoms affected the optical properties of the epitaxial structures. Investigation of suitable GITs in MOCVD can effectively control the wavelength of the GaAsP/InAlGaAs active region and improve interface quality, thereby further enhancing the QW laser performance.

## Figures and Tables

**Table 1:** Comparison of calculated transition wavelengths and PL emission wavelength peaks of GaAsP/InAlGaAs, along with the content and thickness of active region. The e-lh1 and e-hh1 correspond to the transition wavelengths from the first electron level in the conduction band to the first light hole level and the first heavy hole level in the valence band of the GaAsP QW.

Sample	Peak 1 (nm)	Peak 2 (nm)	e-lh1 (nm)	e-hh1 (nm)	QW Thickness (nm)	QW As Concentration (%)	QB Ga/Al Concentration (%)
A	740.24	721.86	740.37	720.79	6.5	73	47/47
B	743.3	723.39	743.24	722.42	6.9	73	47/48
C	746.35	731.06	746.18	733.04	6.2	77	48/48
D	749.41	738.71	749.89	738.8	6.2	78.5	48/48

**Table 2:** The Gaussian fitting parameters *Eg*(0), *α*, *β* for the peak energy of PL versus temperature in Samples A, B, C, and D.

Sample	Transition	*E_g_*_(0)_ (eV)	*α* (eV/K)	*β* (K)
A	e-lh	1.769	4.955 × 10^−4^	195.396
	e-hh	1.814	4.011 × 10^−4^	84.572
B	e-lh	1.765	5.643 × 10^−4^	252.426
	e-hh	1.824	4.846 × 10^−4^	103.768
C	e-lh	1.738	4.688 × 10^−4^	273.293
	e-hh	1.757	2.672 × 10^−4^	4.728 × 10^4^
D	e-lh	1.741	5.910 × 10^−4^	278.405
	e-hh	1.746	4.742 × 10^−4^	325.895

**Table 3:** The fitting parameters of e-lh1 obtained of the normalized PL integrated intensity: *E_A_*_1_, *E_A_*_2_, *C*_1_, *C*_2_ for Samples A, B, C, D.

Sample	*C*_1_	*E_A_*_1_ (meV)	*C*_2_	*E_A_*_2_ (meV)
A	8.08 × 10^7^	363.83	18.25	40.86
B	1.1 × 10^7^	355.74	14.83	55.39
C	6.95 × 10^10^	565.74	15.20	46.73
D	2.88 × 10^5^	235.82	13.15	34.21

**Table 4:** The fitting parameters obtained from the FWHM of Gaussian fitting components in TDPL: Γ_0_, *σ*, *γ* and *ħω_LO_* for e-lh1 in Samples A, B, C and D.

Sample	Γ_0_ (meV)	*σ* (meV/K)	*γ* (meV)	*ħω_LO_* (meV)
A	19.121	0.019	5.484	49.137
B	16.925	0.011	6.677	17.111
C	19.976	0.021	5.771	37.583
D	15.599	0.011	6.616	23.009

## Data Availability

Data are contained within the article.

## References

[1] Imamoto H., Sato F., Imanaka K., Shimura M. (1989). AlGaAs/GaAs Superlattice Multi-Quantum-Well Laser Diode. Superlattices Microstruct..

[2] Bour D.P., Treat D.W., Thornton R.L., Paoli T.L., Bringans R.D., Krusor B.S., Geels R.S., Welch D.F., Wang T.Y. (1992). Low Threshold GaxIn1−XP/(AlyGa1−Y)0.5In0.5P Strained Quantum Well Lasers. J. Cryst. Growth.

[3] Erbert G., Bugge F., Knauer A., Sebastian J., Thies A., Wenzel H., Weyers M., Trankle G. (1999). High-Power Tensile-Strained GaAsP-AlGaAs Quantum-Well Lasers Emitting between 715 and 790 nm. IEEE J. Sel. Top. Quantum Electron..

[4] Bai Y., Xu Z., Lin Y., Wisdom J., Scholz C., Weiss E.S., Chilla J., Diening A. (2018). AlGaAs-Based Optically Pumped Semiconductor Lasers. Vertical External Cavity Surface Emitting Lasers (VECSELs) VIII.

[5] Mogensen P.C., Hall S.A., Smowton P.M., Bangert U., Blood P., Dawson P. (1998). The Effect of High Compressive Strain on the Operation of AlGaInP Quantum-Well Lasers. IEEE J. Quantum Electron..

[6] Shieh H.M., Fu R.J., Lu T.-C., Chen L.-R. (2000). High-Performance GaInP/AlGaInP Visible Laser Diodes Grown by Multiwafer MOCVD. Laser Diodes and LEDs in Industrial, Measurement, Imaging, and Sensors Applications II; Testing, Packaging, and Reliability of Semiconductor Lasers V.

[7] Kaspari C., Zorn M., Weyers M., Erbert G. (2008). Growth Parameter Optimization of the GaInP/AlGaInP Active Zone of 635nm Red Laser Diodes. J. Cryst. Growth.

[8] Hu H.M., Zhao J., Wang W., Ho J., Kuang L., Liu W. (2019). 12 W High Power InGaAsP/AlGaInP 755 Nm Quantum Well Laser. Chin. Opt. Lett..

[9] Mawsi L.J., Rusli S., Al-Muhanna A., Wade J.K. (1999). Short-Wavelength (0.7 Μm>λ>0.78 Μm) High-Power InGaAsP-Active Diode Lasers. IEEE J. Sel. Top. Quantum Electron..

[10] Al-Muhanna A., Wade J.K., Earles T., Lopez J., Mawst L.J. (1998). High-Performance, Reliable, 730-Nm-Emitting Al-Free Active Region Diode Lasers. Appl. Phys. Lett..

[11] Ono K. (2007). Masayoshi Takemi Anomalous Behavior of Phase Separation of InGaAsP on GaAs Substrates Grown by MOVPE. J. Cryst. Growth.

[12] Ono K., Takemi M. (2008). Investigation of Anomalous Optical Characteristics of InGaAsP Layers on GaAs Substrates Grown by Metalorganic Vapor Phase Epitaxy. J. Appl. Phys..

[13] Ono K., Takemi M., Fujiwara Y. (2008). Metalorganic Vapor Phase Epitaxial Growth Parameter Dependence of Phase Separation in Miscibility Gap of InGaAsP. J. Appl. Phys..

[14] Konaka Y., Ono K., Terai Y., Fujiwara Y. (2010). Coexistence Properties of Phase Separation and CuPt-Ordering in InGaAsP Grown on GaAs Substrates by Organometallic Vapor Phase Epitaxy. J. Cryst. Growth.

[15] Knauer A., Bugge F., Erbert G., Wenzel H., Vogel K., Zeimer U., Meyers M.M. (2000). Optimization of GaAsP/AlGaAs-Based QW Laser Structures for High Power 800 Nm Operation. J. Electron. Mater..

[16] Knauer A., Erbert G., Wenzel H., Bhattacharya A., Bugge F., Maege J., Pittroff W., Sebastian J. (1999). 7W CW Power from Tensile-Strained GaAsyP1-y/AlGaAs (=735 nm) QW Diode Lasers. Electron. Lett..

[17] Sebastian J., Beister G., Bugge F., Buhrandt E., Erbert G., Hansel H.G., Hulsewede R., Knauer A., Pittroff W., Staske R. (2000). High-Power 810-nm GaAsP-AlGaAs Diode Lasers with Narrow Beam Divergence. IEEE J. Sel. Top. Quantum Electron..

[18] Sumpf B., Beister G., Erbert G., Fricke J., Knauer A., Pittroff W., Ressel P., Sebastian J., Wenzel H., Tra G. (2001). 2 W Reliable Operation of =735 nm GaAsP/AlGaAs Laser Diodes. Electron. Lett..

[19] Sumpf B., Hu R., Erbert G., Dzionk C., Fricke J., Knauer A., Pittroff W., Ressel P., Sebastian J., Wenzel H. (2002). High-Brightness 735 Nm Tapered Diode Lasers. Electron. Lett..

[20] Wang B., Zhou L., Tan S., Liu W., Deng G., Wang J. (2024). 71% Wall-Plug Efficiency from 780 Nm-Emitting Laser Diode with GaAsP Quantum Well. Opt. Laser Technol..

[21] Ota K., Yaguchi H., Onabe K., Ito R., Shiraki Y. (1994). Effect of Growth Interruption on the Interface Flatness in Metalorganic Vapor Phase Epitaxy-Grown GaAs/GaAsP Heterostructures. J. Cryst. Growth.

[22] Antolini A., Fornuto G., Papuzza C., Soldani D., Taiariol F. (1996). Growth and Characterization of InGaAsP/InGaAs MQW Structures Grown by MOCVD. Technique. Mater. Sci. Forum.

[23] Pan J., Huan B., Zhang X., Yue J., Yu Y., Wei J. (2004). Effect of Growth Interruption and Strain Buffer Layer on PL Performance of AlGaAs/GaAs/InGaAs Quantum Well for 1065 nm Wavelength Lasers. Rare Met..

[24] Demir I., Elagoz S. (2016). Interruption Time Effects on InGaAs/InAlAs Superlattices of Quantum Cascade Laser Structures Grown by MOCVD. Superlattices Microstruct..

[25] Bru-Chevallier C., Baltagi Y., Guillot G., Hong K., Pavlidis D. Impact of Growth Interruption on Interface Roughness of MOCVD Grown InGaAs/InAlAs Studied by Photoreflectance Spectroscopy. Proceedings of the Compound Semiconductors 1997. Proceedings of the IEEE Twenty-Fourth International Symposium on Compound Semiconductors.

[26] Strömberg A., Omanakuttan G., Liu Y., Mu T., Xu Z., Lourdudoss S., Sun Y.-T. (2020). Heteroepitaxy of GaAsP and GaP on GaAs and Si by low pressure hydride vapor phase epitaxy. J. Cryst. Growth.

[27] Wagner J., Peter M., Winkler K., Bachem K.-H. (1998). Interfacial Intermixing and Arsenic Incorporation in Thin InP Barriers Embedded in In0.53Ga0.47As. J. Appl. Phys..

[28] Spurdens P.C., Taylor M.R., Hockly M., Yates M.J. (1991). The Influence of Growth Conditions on the Planarity of MOVPE Grown GaInAs(P) Interfaces. J. Cryst. Growth.

[29] Schwedler R., Gallmann B., Wolter K., Kohl A., Leo K., Kurz H., Juillaguet S., Massone E., Camassel J., Laurenti J.P. (1993). Interface Characterization of Strained InGaAs/InP Quantum Wells after a Growth Interruption Sequence. Appl. Surf. Surf. Sci..

[30] Jordan A.S., Robertson A. (1994). Thermodynamic Analysis of AsH3 and PH3 Decomposition Including Subhydrides. J. Vac. Sci. Technol. A.

[31] Zhong L., Ma X. (2008). Photoluminescence Properties of Tensile-Strained GaAsP/GaInP Single Quantum Wells Grown by Metal Organic Chemical Vapor Deposition. J. Appl. Phys..

[32] Bertolet D.C., Hsu J.-K., Lau K.M. (1988). Tailoring of Hole Eigenenergies in Strained GaAsP/AlGaAs Single Quantum Wells Grown by Atmospheric Pressure Organometallic Chemical Vapor Deposition. Appl. Phys. Lett..

[33] Nakano T., Shioda T., Abe E., Sugiyama M., Enomoto N., Nakano Y., Shimogaki Y. (2008). Abrupt InGaP∕GaAs Heterointerface Grown by Optimized Gas-Switching Sequence in Metal Organic Vapor Phase Epitaxy. Appl. Phys. Lett..

[34] Seifert W., Liu X., Samuelson L. (1993). Influence of Arsenic Adsorption Layers on Heterointerfaces in GaInAs/InP Quantum Well Structures. Appl. Phys. Lett..

[35] López-Escalante M.C., Gabás M., García I., Barrigón E., Rey-Stolle I., Algora C., Palanco S., Ramos-Barrado J.R. (2016). Differences between GaAs/GaInP and GaAs/AlInP Interfaces Grown by Movpe Revealed by Depth Profiling and Angle-Resolved X-Ray Photoelectron Spectroscopies. Appl. Surf. Sci..

[36] Kúdela R., Kučera M., Olejnı B., Eliáš P., Hasenöhrl S., Novák J. (2000). Formation of Interfaces in InGaP/GaAs/InGaP Quantum Wells. J. Cryst. Growth.

[37] Zhang X.B., Ryou J.H., Dupuis R.D., Walter G., Holonyak N. (2006). Metalorganic Chemical Vapor Deposition Growth and Characterization of InGaP/GaAs Superlattices. J. Electron. Mater..

[38] Hou C., Zou Y., Wang H., Wang X., Xu Y., Wang Q., He Z., Fan J., Shi L., Xu L. (2019). Tailoring Strain and Lattice Relaxation Characteristics in InGaAs/GaAsP Multiple Quantum Wells Structure with Phosphorus Doping Engineering. J. Alloys Compd..

[39] Fan H.Y. (1951). Temperature Dependence of the Energy Gap in Semiconductors. Phys. Rev..

[40] Dong H., Sun J., Ma S., Liang J., Lu T., Liu X., Xu B. (2016). Influence of Substrate Misorientation on the Photoluminescence and Structural Properties of InGaAs/GaAsP Multiple Quantum Wells. Nanoscale.

[41] Liu L., Wang L., Li D., Liu N., Li L., Cao W., Yang W., Wan C., Chen W., Du W. (2011). Influence of Indium Composition in the Prestrained InGaN Interlayer on the Strain Relaxation of InGaN/GaN Multiple Quantum Wells in Laser Diode Structures. J. Appl. Phys..

[42] Yuan H., Li L., Zhang J., Li Z., Zeng L., Wang Y., Qu Y., Ma X., Liu G. (2019). Optical Properties Improvement of InGaAs/GaInP Single Quantum Well Grown by Metal-Organic Chemical Vapor Deposition. Optik.

[43] Mickevičius J., Nomeika K., Dmukauskas M., Kadys A., Nargelas S., Aleksiejūnas R. (2021). Comparison of Growth Interruption and Temperature Variation Impact on Emission Efficiency in Blue InGaN/GaN MQWs. Vacuum.

[44] Fang Y., Wang L., Sun Q., Lu T., Deng Z., Ma Z., Jiang Y., Jia H., Wang W., Zhou J. (2015). Investigation of Temperature-Dependent Photoluminescence in Multi-Quantum Wells. Sci. Rep..

[45] Rudin S., Reinecke T.L. (1990). Temperature-Dependent Exciton Linewidths in Semiconductor Quantum Wells. Phys. Rev. B.

